# Household food insecurity access scale and dietary diversity score as a proxy indicator of nutritional status among people living with HIV/AIDS, Bahir Dar, Ethiopia, 2017

**DOI:** 10.1371/journal.pone.0199511

**Published:** 2018-06-28

**Authors:** Foziya Mohammed Hussein, Aragaw Yimer Ahmed, Oumer Sada Muhammed

**Affiliations:** 1 Department of Public Health, College of Medicine and Health Science, Wollo University, Dessie, Ethiopia; 2 Department of Medicine, College of Medicine and Health Science, Wollo University, Dessie, Ethiopia; 3 Department of Pharmacology and Clinical Pharmacy, School of Pharmacy, College of Health Sciences, Addis Ababa University, Addis Ababa, Ethiopia; College of Agricultural Sciences, UNITED STATES

## Abstract

**Background:**

Both household food insecurity and household dietary diversity have been found reliable in describing the dietary intake of a population. However, it had not been proven as reliable instrument for assessing nutritional status of individuals in a clinical context. There has been a need for evidence on the validity of using proxy and easy dietary indicators for nutritional status.

**Method:**

A facility based cross sectional study design was employed on 423 people with *HIV* infection visiting all ART clinics in Bahir Dar, North Ethiopia. Nutritional status was determined by computing *BMI*. Food insecurity was assessed using household food insecurity access scale. Dietary diversity was measured using a tool adopted from Food and Nutrition Technical Assistance Project. Data were entered to Epidata version 3.1and analyzed by *SPSS* version 20. Reliability analysis, sensitivity and specificity analysis were determined.

**Result:**

The sensitivity of the household food insecurity access scale and dietary diversity score was 87.9% and 79.8%, respectively, while their specificity was 56.2% and 70.2%. The *AUC* at 95% CI for the household food insecurity access scale and household dietary diversity score were 73.4 (68.4–78.4) and 73.1 (68.1–78.2) while their cut of point that maximized their sensitivity and specificity was 1 and 6 respectively. Household food insecurity access scale and household dietary diversity score were found to be reliable tools with a Cronbach’s Alpha of 0.926 and 0.799, respectively.

**Conclusion:**

In assessing under nutrition among *PLHIV* especially in limited resource settings, both the household food insecurity access scale and household dietary diversity score were found valid and reliable proxy indicators for measuring nutritional status.

## Background

Food security exists when “all people, at all times, have physical and economic access to sufficient, safe and nutritious food to meet their dietary needs and food preferences for an active and healthy life[1].

The dietary diversity scores consist of a simple count of food groups that a household or an individual has consumed over the past 24 hours[2]. Household dietary diversity score (*HDDS*) is used as a proxy measure of the socio-economic level of the household[3].

Measurement of dietary intake is complex and the most appropriate measurement method will depend on: the objectives of the surveillance; the type of data required; available resources and the population of interest. All of these factors must be considered carefully before selecting a dietary assessment tool [4]. Reliable assessment of nutritional status is essential for identifying nutritional issues and at-risk groups in a given population for the development of dietary intervention programs and for monitoring the efficiency of such interventions[5].

In general, conditions of food insecurity are believed to affect all household members, although not necessarily in the same way. By contrast, under nutrition is affect individuals-some members of the household may be under nourished while others are not. Then, when the household food insecurity access scale (*HFIAS*) classifies a household into severely food insecure with under nutrition, what it shows us at least some member, or a member, of the household are experiencing under nutrition due to inadequacy of food access, but not all members, because this scale has been found reliable for describing the status of a population. It has not yet been proven reliable for assessing an individual nutritional status [6].

There is still a lack of consensus about what dietary diversity really is and what it reflects. Experience from the developing world is scant for the recall period, number and composition of food groups to include in the score and best cut-off for creating a dichotomous indicator. Due to limited research and lack of uniformity for cut-off points this study had a contribution in validating both *HFIAS* and dietary diversity score (*DDS*) and developing appropriate cut-off point by nutritional status as a benchmark on People live with HIV/AIDS (*PLHIV*) and identify simple and reliable proxy indicator that used for early detection of a problem on nutritional vulnerable groups[7].

In addition nutritional assessment through dietary methods used for screening and provides the basis for appropriate counseling and decision making about the need for interventions such as food support. Valid and reliable dietary methods that indirectly measure or alarm nutritional status, is help for the detection of nutritionally vulnerable population groups like *PLHIV* and the implementation of effective strategies addressing these issues in resource limited situation. And also scientific evidence is needed to choose the best and easy dietary method which further contributes for adherence to *ART* and other treatment outcomes.

## Methods

### Study setting and sampling

A facility-based, cross-sectional study design was employed in February, 2017 in Bahrdar city administration, a capital of Amhara regional state, North- western Ethiopia. The study included all of the available ten *ART* clinics, of which seven were governmental and three private. The study population were all adults people (age>18 years) live with *HIV* infection attended Bahir Dar *ART* clinics during the study period. The sample size (423 *PLHIV*)for the study was determined assuming a 50% of proportion, a 95% confidence level, a 5% margin of error and an expectation of 10% non-response rate.Using proportional to population size the required sample size was allocated for all clinics.

### Data collection

Household food insecurity access was measured using items from the Household Food Insecurity Access Scale (*HFIAS*) of FAO-FANTA. The *HFIAS* consists of 9 items specific to an experience of food insecurity occurring within the previous four weeks. Each respondent were indicate whether they had encountered the items due to lack of food or money to buy food in the last one month. Endorsed a standard scoring procedure was used with 1 point for occurrence and 0 for non-occurrence. The frequency scores were ranged from 0 to 3, while 0 was the score for non-occurrence, 1 for rarely (once or twice in the past four weeks), 2 for sometimes (three to ten times in the past four weeks), and 3 for often (more than ten times in the past month). For the purpose of this paper, we were used the total score (9-items based on the frequency score). A total score of 27 represents the most food-insecure household whereas a lower score represents a more food-secure household [8].

Data on household dietary diversity were collected using a 24-hour recall method and information was entered into the *HDDS* sheet. The *HDDS* captures dietary diversity in a normal 24-hour period by the household as a whole and not a single member. Food consumed outside the home that was not prepared in the home was not included. A set of 12 food groups were used to guide the scoring as per the food items consumed, with 0 being the minimum score and 12 as the maximum [3, 9].

Anthropometric measurements height was taken by using a stadiometer when the respondent was standing straight and without shoes. The readings were taken at the apex of the head with 0.1 cm accuracy[10]. Weight was taken by using Seca and subjects were asked to remove footwear, heavy clothes and heavy items from their pockets. Measurements were taken to 0.1 kg accuracy. All measurements were taken two times and the mean was recorded[11].To determine individual’s nutritional status, *BMI* was determined by their body mass index calculated using quetlet formula[6].

### Data analysis

We used EpiData Version 3.1 for the data entry, and the data was exported to Statistical Package for Social Sciences20.0 (SPSS) for cleaning and further analysis. Reliability analysis was done for all items of *HFIAS* and *HDDS*. Cronbach’s Alpha was used to present the result and interpreted as very high reliability = 0.90 and above, high reliability = 0.70 to <0.90, moderate reliability = 0.50 to <0.70, low reliability = 0.30 to <0.50. If the items total correlation > 0.3 the item had good, between 0.1 and 0.3 fair, and < 0.1 poor item total correlation [12].

*HFIAS* and *HDDS* were evaluated for sensitivity and specificity using nutritional status as the benchmark by Receiver operating characteristic (*ROC*) curves. Sensitivity and specificity analysis were performed to quantify the accuracy of the tools to correctly classify person nutritional status and then to determine the *HDDS* and *HFIAS* cut-off point that maximized sensitivity and specificity. A proxy indicator strongly associated with nutritional status had high specificity and high sensitivity. In a *ROC* curve the true positive rate (Sensitivity) was plotted in function of the false positive rate (100-Specificity) for different cut-off points of the test variable (number of scores) based on the results of nutritional status as gold standard. Each point on the ROC curve represents a sensitivity/specificity pair corresponding to a particular decision threshold. A test with perfect discrimination (no overlap in the two distributions) had a *ROC* curve that passes through the upper left corner (100% sensitivity, 100% specificity). Therefore the closer the *ROC* curve is to the upper left corner, the higher the overall accuracy of the test.

Area under *ROC* curve (*AUC*) used as an indication of the predictive power or the accuracy of the proxy indicator to correctly classifies nutritional status. If AUC is 0.50 there are no acceptable combinations of sensitivity and specificity can be found. A perfect classification by the proxy indicator would result in an *AUC* of 1.00. As a general rule of thumb, an *AUC* below 0.60 is considered not acceptable, above 0.70 is good, higher than 0.80 is very good, and greater than 0.90 is excellent[13].

## Results

### Household food insecurity and household dietary diversity of PLHIV and nutritional status

Households who experienced anxiety and uncertainty related to food availability in the past four prior to data collection week were 195(46.1%). Those reported consumption of food which was insufficient in terms of quality and quantity were 225(53.2%) and 102(24.1%) respectively. Half of the study participants 214 (50.6%) also reported inability to eat preferred foods. Nearly a quarter 99(23.4%) reported consumption of reduced size of meals and 71(16.8%) households reported reduced number of meals intake. Few 24(5.7%) households reported moments of lack of any kind of food to eat in the past four weeks 17(4.0). The overall food insecurity in the setting was 239 (56.5%). Regarding dietary diversity Cereals and legume intake 24hour prior to the survey were 419(99.1), 335(83.9%) respectively. Vegetable and fruit intake was 287(67.8%) and 148(35.0%) respectively. Household’s achievement of optimal dietary diversity was 55.6%. The mean (*SD*) of *BMI* was 19.8 (2.4) kg/m2.Under nutrition was 29.3%while few 2.4% had over nutrition.

### Reliability of household food insecurity access scale and household dietary diversity score

*HFIAS* showed a cronbach’s Alpha of 0.926.From 9 frequency and occurrence measurement tools if ‘Did you or any household member go to sleep a night hungry because there was not enough food’ and ‘Did you or any household member go a whole day and night without eating anything’ deleted the cronbach’s alpha would go up to 0.927, 0.927 respectively. *HDDS* had yield a cronbach’s Alpha of 0.799. Cereals had poor item total Correlation 0.051. If cereals, legumes, nuts and seeds, and spices condiments beverages deleted the cronbach’s Alpha would raise to 0.805, 0.807 and 0.804 respectively.

### Sensitivity and specificity of HFIAS and HDDS

**Fig 1:**
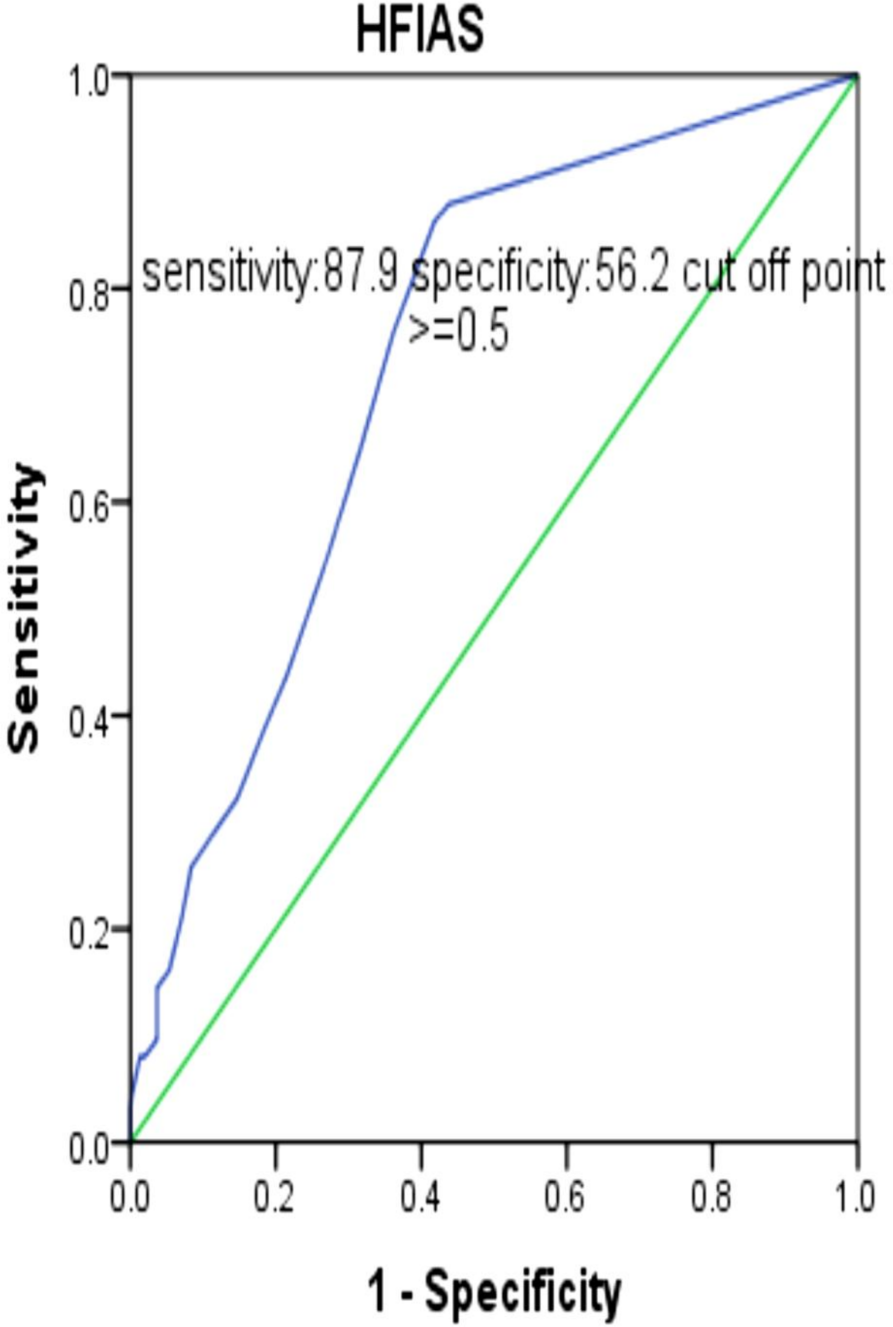
Receiver operating characteristic curve of HFIAS by using nutritional status as a bench mark of PLHIV in all Bahir Dar ART clinics, 2017.

*HFIAS* is a good proxy indicator for measuring nutritional status of *PLHIV* (*AUC* = 73.4%, 95% *CI* = 68.4–78.4).The sensitivity and specificity of *HFIAS* by using nutritional status as a bench mark with optimal criteria of 0.5 and above were 87.9% and 56.2%, respectively (Fig 1).

The sensitivity and specificity of *HFIAS* if the score is 1.5 and above were 86.3% and 58.2%respectively. The sensitivity and specificity of HFIAS if the score is 2.5 and above were 75.8% and 63.9%respectively. The specificity of *HFIAS* if the score is 17.5 and above was 100% (Table 1).

**Table 1:** Results of receiver operating characteristic curve analyses of household food insecurity access scale with sensitivity and specificity of PLHIV in all ART clinics of Bahir Dar, 2017.

Criterion	Sensitivity	Specificity
> = -1.00	1.00	0.00
> = .50	87.9	56.2
> = 1.50	86.3	58.2
> = 2.50	75.8	63.9
> = 3.50	65.3	68.2
> = 4.50	54.8	72.9
> = 5.50	43.5	78.6
> = 6.50	38.7	81.6
> = 7.50	32.3	85.3
> = 8.50	28.2	89.3
> = 9.50	25.8	91.6
> = 10.50	21.0	93.0
> = 11.50	16.1	94.6
> = 12.50	14.5	96.3
> = 13.50	9.7	96.3
> = 14.50	8.1	98.0
> = 15.50	8.1	98.7
> = 16.50	4.8	99.7
> = 17.50	3.2	1.00
> = 18.50	2.4	1.00
> = 21.50	8.0	1.00
> = 25.00	0.00	1.00

**Fig 2:**
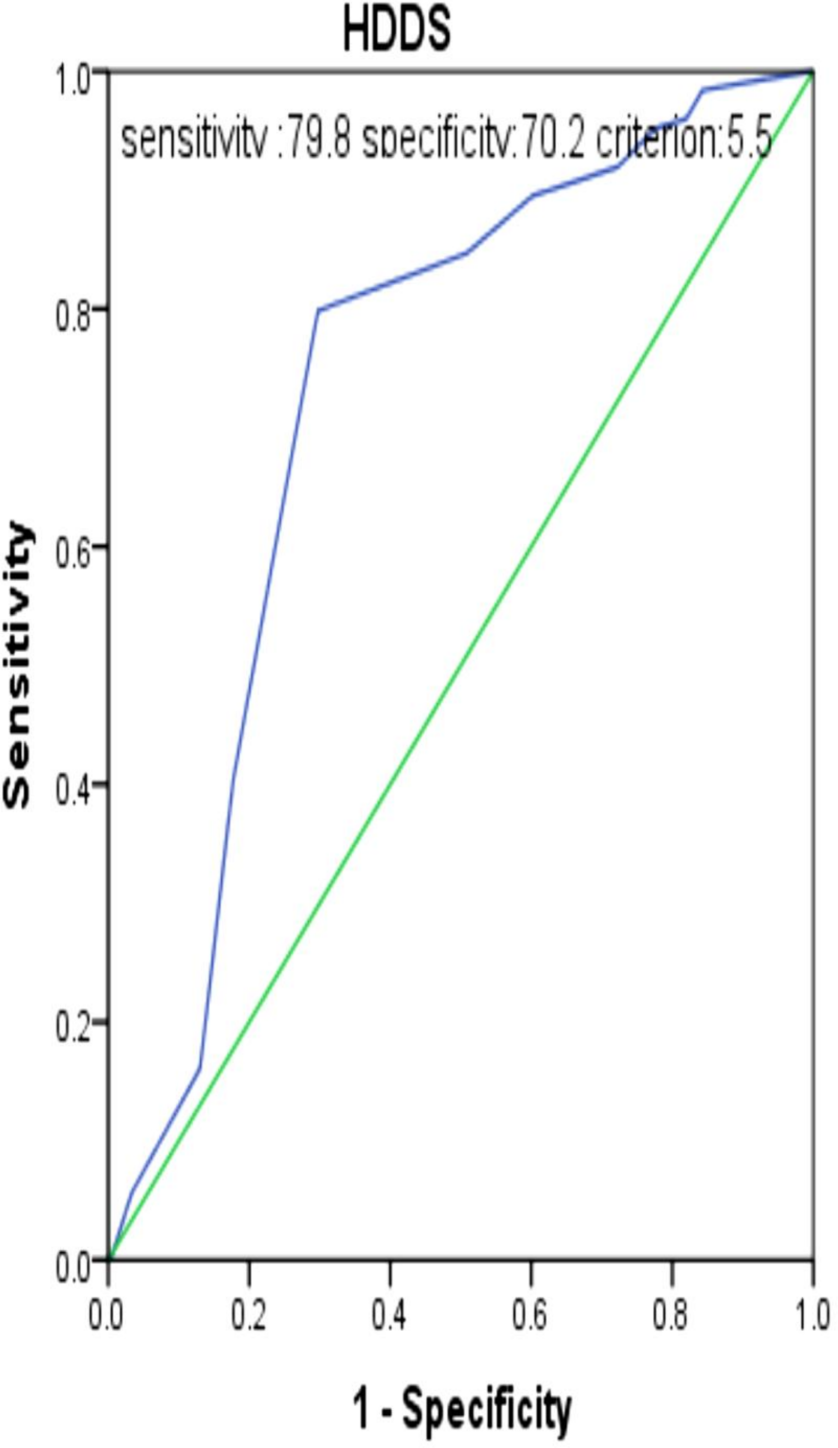
Receiver operating characteristic curve of HDDS by using nutritional status as a bench mark of PLHIV in all Bahir Dar ART clinics, 2017.

*HDDS* is a good proxy indicator for measuring nutritional status of *PLHIV* (*AUC* = 73.1%, 95% *CI* = 68.1–78.2). The sensitivity and specificity of HDDS by using nutritional status as a bench mark with optimal criteria of 5.5 and below were 79.8% and 70.2%, respectively (Fig 2).

The sensitivity and specificity of HDDS to detect under nutrition if the score was 4.5 and below were 40.3% and 82.3%, respectively. The sensitivity and specificity of *HDDS* to detect under nutrition if the score was 6.5 and below were 84.7% and 49.2%, respectively (Table 2).

**Table 2:** Results of receiver operating characteristic curve analyses of household dietary diversity score with sensitivity and specificity of PLHIV in all ART clinics of Bahir Dar, 2017.

Criterion	Sensitivity	Specificity
< = 0.00	0.00	1.00
< = 1.50	0.00	99.7
< = 2.50	5.6	96.7
< = 3.50	16.1	87.0
< = 4.50	40.3	82.3
< = 5.50	79.8	70.2
< = 6.50	84.7	49.2
< = 7.50	89.5	39.8
< = 8.50	91.9	27.8
< = 9.50	95.2	22.4
< = 10.50	96.0	18.4
< = 11.50	98.4	15.7
< = 13.00	1.000	0.00

## Discussion

In the analysis, household food insecurity access scale was found to have high reliability with cronbach’s Alpha of 0.92. From the 9 items of *HFIAS* removing severity indicating items number 8^th^ and 9^th^did not change the value of cronbach’s Alpha. This finding is in line with the *UNICEF* study in Indian [14]and Butajira, Ethiopia [15]. Similarly, Household dietary diversity measurement tool was also found reliable with cronbach’sAlphaof0.79. Among 12 items cereals had low item total correlation if deleted it couldn’t derange cronbach’s Alpha so all items were reliable for measuring dietary diversity of households.

*HFIAS* can be used as aproxy indicator for detecting nutritional status of *PLHIV* with *AUC* of 73.4. *HFIAS* had good sensitivity (87.9%) but low specificity (56.2%) by optimal criteria of 1. This indicates that 87.9 percent of positive outcomes correctly classify by using cutoff point of greater than or equal to 1, but only 56.2% of negative outcomes were correctly classified by using 1 as a cutoff point from a total of 27 scores.

*HDDS* was also found as good proxy indicator for measuring nutritional status of *PLHIV* with *AUC* of 73.1% or the overall accuracy of the tool correctly classifies nutritional status was 73.1%. The true positive rate of *HDDS* was 79.8% or 79.8% of positive outcomes were correctly classified by less than six food group as a cutoff point from a total of 12 food groups and 70.2 percent of negative outcomes were correctly classified by this cutoff point.

*HFIAS* and *HDDS* were proxy indicators for nutritional status but not a diagnostic or direct indicator for nutritional status that is why the accuracy of the tools correctly classifies nutritional status were lies in fair range between 70% and 80%. *HFIAS* was a sensitive (87.9%) proxy indicator as compared to *HDDS*, but *HDDS* is a specific (70.2%) as compared to *HFIAS*, it is due to the recall period for *HFIAS* 30 days for *HDDS* 24 hour and *HDDS* more focus on the dietary intake of household members where as *HFIAS* tools combine both dietary intake and food access items [3, 8]. Generally the predictive power of *HFIAS* is slightly higher than *HDDS*.

## Conclusion

Both *HFIAS* and *HDDS* were found reliable for measuring nutritional status of *PLHIV*. In assessing under nutrition among *PLHIV* especially in limited resource setting, both Household food insecurity access scale and household dietary diversity score were found valid and reliable proxy indicator for measuring nutritional status.

## Abbreviations

AIDS: Acquired Immune Deficiency Syndrome
ART: Anti-Retroviral Therapy
AUC: Area Under ROC Curve
BMI: Body Mass Index
CI: Confidence Interval
FAO: Food and Agriculture Organization
HDDS: Household Dietary Diversity Score
HFIAS: Household food insecurity access scale
HIV: Human Immune Deficiency Virus
PLHIV: People Living With Human Immune deficiency Virus
ROC: Receiver Operating Characteristic
SPSS: Statistical Package for Social Sciences
UNICEF: United Nations Children’s Fund
